# Automated planning of whole breast irradiation using hybrid IMRT improves efficiency and quality

**DOI:** 10.1002/acm2.12767

**Published:** 2019-11-19

**Authors:** Bingqi Guo, Chirag Shah, Ping Xia

**Affiliations:** ^1^ Department of Radiation Oncology Taussig Cancer Institute Cleveland Clinic Cleveland OH USA

**Keywords:** auto‐segmentation, auto planning, breast IMRT, whole breast irradiation

## Abstract

**Purpose:**

To develop an automated workflow for whole breast irradiation treatment planning using hybrid intensity modulated radiation therapy (IMRT) approach and to demonstrate that this workflow can improve planning quality and efficiency when compared to manual planning.

**Methods:**

The auto planning framework was built based on scripting with MIM and Pinnacle systems. MIM workflows were developed to automatically segment normal structures and targets, identify landmarks for beam placement, select beam energies, and set beam configurations. Pinnacle scripts were generated from the MIM workflow to create hybrid IMRT plans automatically. Each hybrid IMRT plan included two prescriptions: a three‐dimensional (3D) prescription consisted of two open tangent beams, and an IMRT prescription consisted of two step‐and‐shoot IMRT beams. The 3D prescription delivered a full prescription dose to the maximum dose point, and the IMRT prescription was optimized to deliver a uniform dose to the entire breast while sparing dose to the normal structures. For 30 patients, the auto plans were compared with clinically accepted manual plans using the paired sample *t*‐test.

**Results:**

The auto planning process took approximately 8 min to complete. The mean dice coefficients between auto‐segmentation and manual contours were 0.98, 0.94 and 0.88 for the lungs, heart, and PTVeval_Breast, respectively. The MUs of the auto plans was on average 13% higher than that of the manual plans. Auto planning improved plan quality significantly: percentage volume receiving 95% of the prescription dose (V95%) of the PTVeval_Breast increased from 91.5% to 93.2% (*P* = 0.001), V105% of the PTVeval_Breast decreased from 7.2% to 1.2% (*P* = 0.013), V20Gy of the ipsilateral lung decreased from 13.1% to 10.4% (*P* = 0.001) and mean heart dose for left‐sided breast patients decreased from 1.2 Gy to 0.9 Gy (*P* < 0.001).

**Conclusion:**

An automated treatment planning process can make the planning process efficient with improved plan quality.

## 1 INTRODUCTION

Whole breast irradiation (WBI) using tangential fields is an established technique for adjuvant radiation therapy as part of breast‐conserving treatment of early‐stage breast cancer.^1^ The planning goals of WBI include delivering a uniform dose to the breast tissue while minimizing dose to the lungs and the heart. Based on patient‐specific anatomy, planners manually select tangent beam configuration (gantry angles and collimator angles) and beam energies, design the beam apertures (including segments), and set beam weights through a time‐consuming trial and error approach. Planning time was on average 39 min (range: 15–70 min) in a 20 patient study.^2^ Several auto and semi‐auto planning techniques have been developed to improve the efficiency of each step of WBI treatment planning. These steps included automatically segmenting the treatment targets and normal structures,^3^, ^4^, ^5^, ^6^ automatically selecting beam angles,^7^ optimizing wedge angles for tangent beams,^8^ optimizing segment shapes and weights,^3^, ^9^, ^10^ and automatically creating an inverse planned intensity modulated radiation therapy (IMRT) plan.^4^, ^11^, ^12^

Automation can reduce planning time with comparable or better plan quality. Zhao et al. proved that automatic beam angle selection reduced the volume of heart receiving 5 Gy and volume of ipsilateral lung receiving 10 Gy.^7^ Mitchell et al. used auto‐segmentation software to contour critical structures and scripting in the treatment planning system to generate beam segments from isodose lines and optimize segment weights.^3^ They concluded that auto contours agreed closely with clinician delineation and scripted treatment plans demonstrated equivalence with their clinical counterparts with modest deduction in planning time. Purdie et al. developed a fully automatic planning technique for tangent step‐and‐shoot IMRT.^4^ This technique used radio‐opaque markers placed at CT simulation to determine the beam geometry and generate whole breast volume for inverse IMRT optimization. For the 158 patients studied, the mean planning time was 6.8 min. Ninety‐nine percent of the auto plans were deemed clinically acceptable, and 87% were deemed clinically improved or equal to manual plans. Purdie’s method was applied clinically to over 1600 patients and was shown to reduce plan rejection rates.^8^

We adapted Purdie's method to our clinical practice and improved the workflow to overcome some limitations of the original technique: (a) we allow flexibility in determining treatment boundaries and do not require wiring the patient in a specific way; (b) we allow the use of mixed energies and perform beam weight optimization automatically; (c) we automate and standardize the use of heart and lung blocks; and (d) we use a new hybrid IMRT technique which can maximize the weight of open beam to improve delivery efficiency and robustness. In this study, we described our auto planning workflow and compared plans created with this workflow with clinical plans for volume delineation, beam arrangement, planning parameters, plan quality and delivery robustness.

## 2 MATERIALS AND METHODS

### 2.1 Patients

This institutional review board (IRB) approved retrospective study included 30 patients randomly selected from patients treated at our institution with tangential field‐in‐field WBI between November 2016 and November 2018. In this cohort, 10 patients were left breast cancer treated with deep inspiration breath hold (DIBH), 10 patients were left breast treated without DIBH, and ten patients were right breast cancer treated. The clinical plans for these patients used 6/10 MV photon with four standard fraction and 26 hypofractionated schemes. All patients were planned using Pinnacle treatment planning system, version 9.10 (Philips Healthcare, Fitchburg, WI) and treated on Truebeam Linear accelerator (Varian Medical Systems, Palo Alto, CA).

### 2.2 Auto planning workflow

**Figure 1:**
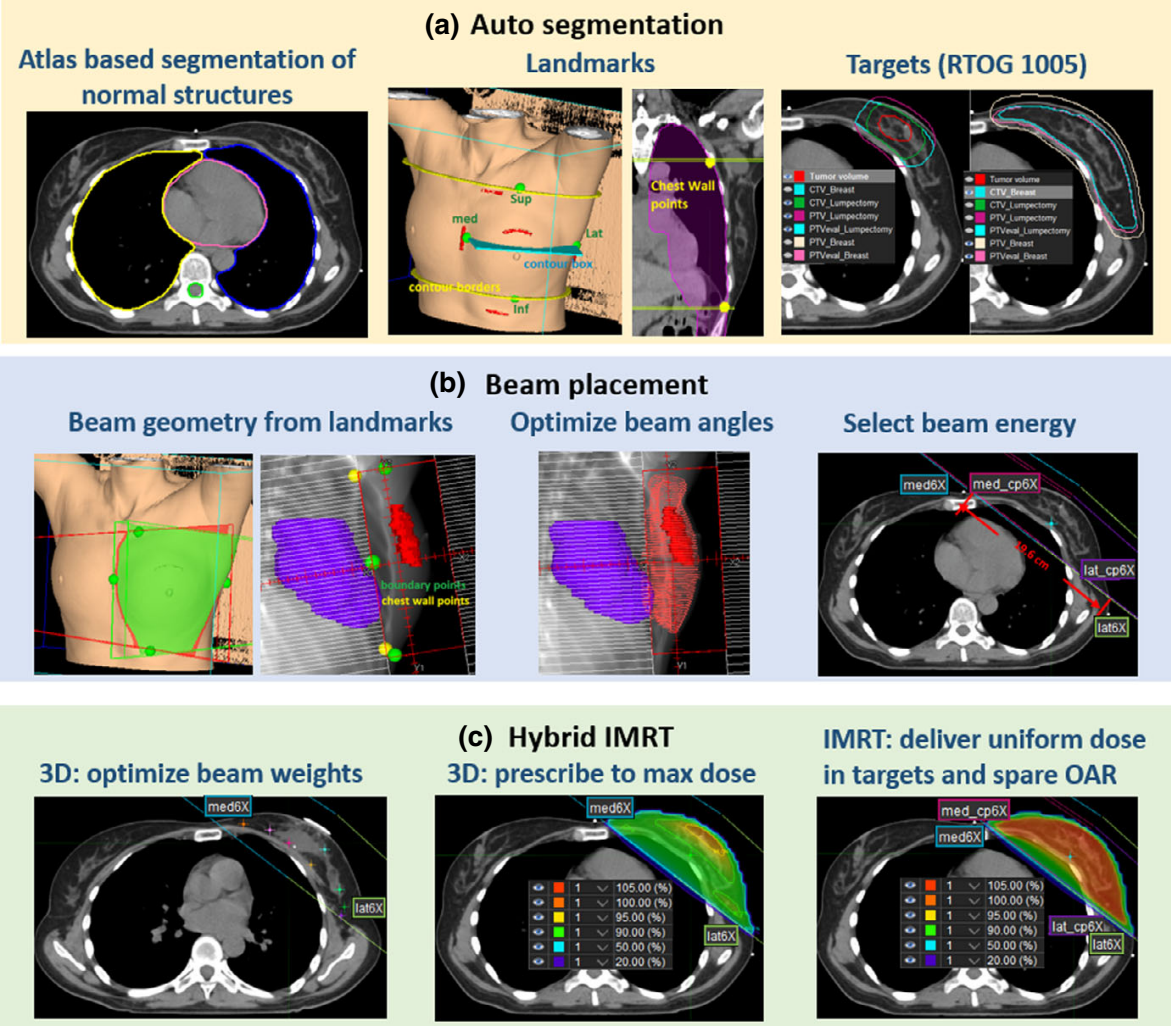
Auto planning workflow. (a) Auto‐segmentation: normal structures including bilateral lungs, heart, and spinal cord were contoured using atlas‐based segmentation; the superior, inferior, lateral and medical boundary landmarks were identified from the “box” and “border” contours or the wires placed on the patient skin. Two chest wall contours were identified from the computed tomography; targets were segmented following RTOG 1005 recommendations. (b) Beam placement: two tangent beams were placed based on the boundary points and chest wall points. The gantry angle, collimator angle, and jaw positions were then optimized to maximize target coverage and minimize normal lung and heart volume in the beam. Beam energy was selected based on the maximal separation; (c) Hybrid IMRT: the automatic breast plan includes two prescriptions: a three‐dimensional (3D) prescription with two static tangent beams and an intensity modulated radiation therapy (IMRT) prescription with two step‐and‐shoot tangent beams. The beam weightings of the 3D prescription were optimized using dose points selected uniformly inside PTVeval_breast. The 3D prescription delivers full prescription dose to the maximum dose. The IMRT prescription was optimized to deliver uniform dose to breast and reduce dose to lungs and heart.

For this study, all patients were auto planned using the same dataset, treatment machine, isocenter, and prescription dose as the corresponding clinical plans. Figure 1 shows the auto planning workflow. It consists of the following steps:

- Auto‐segmentation of volumes of interests and beam boundary points

Computed tomography images were sent to the MIM system (MIM Software Inc., Cleveland, OH, USA). An assistant rule had been set up in MIM to automatically perform atlas‐based segmentation for normal structures including bilateral lungs, heart, and spinal cord using an MIM workflow and a breast atlas, which was developed based on 20 patients outside of this study cohort.

Physicians reviewed the CT images to determine the extent of breast tissue to be treated based on wires placed at simulation and clinical judgment. They had the option to redefine the medial and lateral boundaries (using a contour named “box”) and the superior and inferior boundaries (using a contour named “borders”). Another MIM workflow was developed to detect four boundary points: medial, lateral, superior and inferior based on the “box” and “borders” contours if they existed. Otherwise, the wires were automatically detected and use for boundary placement. In addition to the boundary points, two chest wall points, one at the chest wall & superior boundary and one at the chest wall & inferior boundary of the treatment region were placed by the MIM workflow. The boundary points and chest wall points were used to set up beams. The middle panel in Fig. 1(a) illustrates how to define the boundary and chest wall points from contours and CT.

Once beam boundaries were defined, the MIM workflow continued to segment the target volumes following the definitions of RTOG 1005 (Table 1).^13^

- Beam placement and beam parameters optimization

**Table 1:** Target volume generation following Section 2.A and 2.B of RTOG 1005.

Target volume	Definition
*GTV_Lumpectomy*	Drawn by physicians
*CTV_Lumpectomy*	Expand GTV_Lumpectomy by 1 cm in all directions, limited posteriorly to the pectoralis major, anterolaterally to 5 mm from the skin and medially to the midline
*PTV_Lumpectomy*	Expand CTV_Lumpectomy by 7 mm in all directions, excluding the heart
*PTVeval_Lumpectomy*	PTV_Lumpectomy within ipsilateral breast tissue and 5 mm from the skin
*CTV_Breast*	Drawn by physicians, OR, Breast tissue, segmented based on the HU threshold, within the volume defined by boundary points
*PTV_Breast*	Expand CTV_breast by 7 mm in all directions, excluding the heart and not crossing the midline
*PTVeval_Breast*	PTV_breast limited posteriorly to ribs and anterolaterally to 5 mm from the skin
*PTVeval_Breastopt*	The intersection of PTVeval_Breast and the volume under beam contracted 5mm uniformly. This volume was used for optimization only

Two tangent beams were created automatically using a Matlab (the MathWorks, Inc., Natick, Massachusetts, USA) script embedded in the MIM workflow. The gantry angle of the medial beam was set so that the medial and lateral boundary points overlapped each other in the beam's eye view. The collimator angle was set so that X jaws of the beam were parallel with the two chest wall points. The position of the posterior jaw (X1) was placed at the medial and lateral boundary points, the anterior jaw (X2) covered the entire breast volume with 2 cm skin flash, and the superior (Y2) and inferior (Y1) jaw positions were defined at superior and inferior boundary points. The lateral beam was a mirror of the medial beam adjusted for nondivergence posteriorly. The left panel in Fig. 1(b) illustrates how to set up beams based on boundary and chest wall points.

The beam angles and jaw positions were further optimized to cover the targets and reduce the lung and heart volumes in the beam. Because the lateral beam matched to the medial beam, only the medial beam was optimized. The gantry angle of the medial beam was limited to ±10° from its starting angle defined using the boundary points. The collimator angle of the medial beam was also limited to ±10° from the starting angle defined by the chest wall points. For each gantry/collimator angle combination, the beam’s posterior jaw (X1) position was set to cover 95% of the PTVeval_breast and 100% of the tumor bed with 5 mm margin. Both lateral and medial beams were restricted not to cross the midline of the patient. The beam's superior (Y2) and inferior (Y1) jaw positions were set to cover the entire PTVeval_Breast plus 5mm and the superior/inferior boundary points, whichever was larger. The volume of the heart and lungs inside the beam was calculated and an objective function defined in Eq. (1) was minimized.

(1) f=50×%heartvolumeinthebeam+%lungvolumeinthebeam

A ratio 50 was arbitrarily chosen to reflect the importance of reducing heart dose. The combination of gantry angle, collimator angle, and jaw positions which gave the smallest f value was chosen.

After beam geometry optimization, if more than 10 cc heart volume were exposed in the beam, a heart block was added to block all or part of the heart without blocking the lumpectomy cavity with a 5 mm margin. For ipsilateral lung and the normal tissue inferior to the ipsilateral lung, a lung block was added if it did not block any part of PTVeval_Breast with 1 cm margin.

A patient‐specific pinnacle script was created at the end of the MIM workflow for automatic hybrid IMRT planning.

- Hybrid IMRT planning

The hybrid IMRT plan included a 3D prescription and an IMRT prescription. The 3D prescription was associated with a pair of open medial and lateral beams (with heart and lung blocks if used), and the IMRT prescription was associated with two step‐and‐shoot beams using the same beam geometry as the 3D tangent beams. The energy of the beams was defined based on the maximum tangent separation, same as our clinical practice. If the maximum separation of the tangent beams was less than 20 cm, 6 MV was used for both 3D and IMRT prescriptions; for separation great than 23.5 cm, 10 MV was used for both 3D and IMRT prescriptions; for separation between 20 and 23.5 cm, 6 MV was used for the 3D prescription, and 10 MV was used for the IMRT prescription. If the tumor bed volume was within 5 mm from the skin, 6X was used for the 3D prescription regardless of the tangent separation. The 3D prescription delivered a full prescription dose to the maximum dose point. The weights of the medial and lateral 3D beams were initially set to be equal and then optimized (beam weight optimization) based on dose points uniformly placed within the breast tissue. IMRT prescription was optimized to increase homogeneity and reduce dose to the heart and lungs. Table 2 lists the optimization criteria. The IMRT plan allowed a maximum of 20 segments (10 segments for breath hold plans to reduce treatment time). Minimum segment area was set to 10 cm^2^ to force the use of large segments. The “use current jaw as max” feature was checked so that the IMRT segments did not exceed the edges of open beams.

**Table 2:** Intensity modulated ratiation therapy (IMRT) optimization criteria for hybrid IMRT planning.

Volume	Optimization criteria	Weight
*PTVeval_Breastopt*	Receive a uniform dose of 100% prescription	50
	Maximum dose < 105% of prescription	100
*PTVeval_Lumpectomy*	Minimum dose> 100% prescription	100
*Lungs*	V40% < 15%	5
*Heart*	Max EUD (α = 1) < 1 Gy	1

### 2.3 Comparing auto and clinical plans

The auto‐segmented contours were compared with the clinical contours using the Dice coefficient and mean Hausdorff distance. For a fair plan comparison, the auto contours were discarded, and the clinical contours were used for subsequent beam placement and hybrid IMRT planning.

To compare beam parameters between auto plans and clinical plans, we calculated the absolute differences of gantry and collimator angles, the difference in the posterior jaw (X1), the inferior jaw (Y1) and the superior jaw (Y2). The anterior jaw X2 covered the entire breast with >=2 cm margin in both clinical and auto plans. The auto placed beams were used for hybrid IMRT planning.

To compare the plan quality between auto plans (with clinician derived volumes) and clinical plans, target coverage PTVeval_Breast V95 and PTVeval_Lumpectomy V95, high dose volume PTVeval_Breast V105, ipsilateral lung receiving 20 and 5 Gy and heart mean dose were compared. Statistical analysis was performed using two‐sided, paired sample t‐test in Excel with significance defined as *P* < 0.05.

## 3 RESULTS

The entire auto planning workflow took approximately 8 min, of which auto‐segmentation took about 3 min (without manual editing), beam placement took about 2 min, and hybrid IMRT planning took about 3 min.

### 3.1 Auto‐segmentation

**Figure 2:**
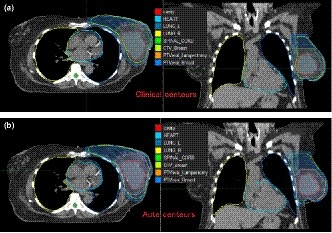
Comparing auto contours (bottom figure) with clinical contours (top figure) for case #1.

The auto‐segmentation achieved good agreement with the clinical contours. Figure 2 shows an example. Table 3 lists the dice coefficients and mean Hausdorff distances between auto contours and clinical contours. The Dice coefficients were more than 0.9, and the mean Hausdorff distances were within 1.5 mm for all normal structures. No manual editing was necessary for the contours of the lungs or spinal cord while only small edits were needed for the heart contours. For targets, dice coefficients of 0.84 and 0.88 were achieved with CTV_breast and PTVeval_breast, respectively.

**Table 3:** Dice coefficients and mean Hausdorff distances between auto‐segmentation and clinical contours for all patients. (mean ± standard deviation).

	Left lung	Right lung	Heart	Spinal cord	CTV_Breast	PTVeval_Breast
Dice coefficient	0.97 ± 0.01	0.98 ± 0.01	0.94 ± 0.03	0.91 ± 0.03	0.84 ± 0.05	0.88 ± 0.05
Mean Hausdorff distance/mm	0.7 ± 0.2	0.7 ± 0.2	1.5 ± 0.8	0.5 ± 0.2	4.4 ± 1.6	3.5 ± 1.7

### 3.2 Auto plan VS clinical plan

**Figure 3:**
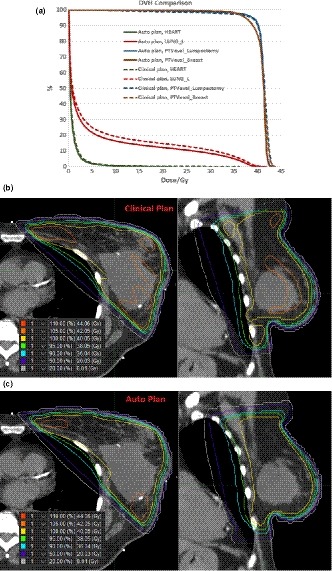
Dose volume histogram (DVH) and isodose comparisons between the auto plan and the clinical plan for case #1. Top figure: DVH comparison; middle figure: isodose distribution of the clinical plan; bottom figure: isodose distribution of the auto plan.

Figure 3 and Table 4 compares plan parameters (beam geometry, energy, and MUs) and plan quality dose volume histogram [(DVH and isodose)] for case #1. The auto plan reproduced the clinical beam set up with slight changes of the gantry (2°) and collimator (4°) angles. Plan monitor unit (MU) increased by 6% as a result of increased intensity modulation. The angle optimization and inverse planned intensity modulation improved the uniformity of dose in the breast and reduced the dose to ipsilateral lung and heart without compromising the target coverage.

**Table 4:** Comparing plan parameters and plan quality between auto plan and clinical plan for case #1.

	Clinical plan	Auto plan
Prescription	2.67 Gy × 15	
Energy	10X	10X
Gantry (medial beam)/°	309	311
Collimator (medial beam)/°	12	8
X1 (posterior jaw)/cm	4.5	4.5
Y1 (inferior jaw)/cm	9	9.5
Y2 (superior jaw)/cm	12	11
Plan MU	312	332
PTVeval_Breast V95/%	93.5	93.3
PTVeval_Lumpectomy V95/%	95.7	96.4
PTVeval_Breast V105/%	17.7	4.7
Lt Lung V20/%	14.7	12.1
Heart Dmean/Gy	1.0	0.8

**Figure 4:**
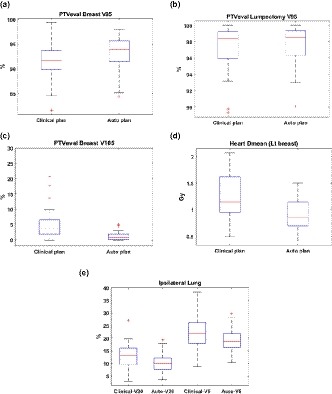
Comparing plan quality between auto plans and clinical plans for all patients.

For all patients, Fig. 4 compares PTVeval_Breast V95, PTVeval_Lumpectomy V95, PTVeval_Breast V105, ipsilateral Lung V20, V5 and Heart Dmean between auto plans and clinical plans. On average, auto plan increased the coverage of PTVeval_Breast V95 from 91.5% to 93.2% (*P* = 0.001), PTVeval_lumpectomy V95 stayed the same (97.1% vs 97.6%, *P* = 0.110), hotspot in breast PTVeval_Breast V105 decreased from 7.2% to 1.2% (*P* = 0.013), ipsilateral lung V20 and V5 decreased from 13.1% to 10.4% (*P* = 0.001) and from 22.4% to 18.7% (*P* < 0.001) respectively, and heart mean dose for left‐sided breast patients decreased from 1.2 to 0.9 Gy (*P* < 0.001). Compared with clinical plans, auto plans improved target coverage, dose uniformity and reduced dose to lung and heart.

Table 5 compares the plan parameters between clinical plans and auto plans. Auto plans reproduced the clinical beam setup with slight changes in gantry (3.6° on average) and collimator (4.5° on average) angles. Jaw positions were the same (average difference <2 mm). MU of auto plans increased by 13% compared with clinical plans. The increased MU was a result of increased intensity modulation. While open beam MUs were almost the same between auto plans and the clinical plans, percentage of MU delivered by open beam decreased from 88% of clinical plans to 76% of auto plans.

**Table 5:** Comparing plan parameters between auto plans and clinical plans for all patients.

	Mean ± SD
Gantry angle/° |auto‐clinical|	3.6 ± 2.6
Collimator angle/°|auto‐clinical|	4.5 ± 2.5
X1/cm auto‐clinical	−0.2 ± 0.5
Y1/cm auto‐clinical	−0.1 ± 0.6
Y2/cm auto‐clinical	0.1 ± 0.9
Total MU auto/clinical	1.13 ± 0.08
Open beam MU auto/clinical	0.97 ± 0.02
Fraction of MU delivered by open beam	Clinical plans: 0.88 ± 0.03 Auto plans: 0.76 ± 0.05

## 4 DISCUSSION

Forward planned, field‐in‐field IMRT is a widely used technique for whole breast irradiation and has been shown to improve cosmetic results and reduce acute side effects.^14^, ^15^ The treatment planning process of field‐in‐field technique is time‐consuming and user dependent. Auto and semi‐auto techniques have been developed to emulate all or parts of the manual planning process, improving planning efficiency and reproducibility.

We adapted and improved previously published auto planning techniques to our clinical practice and developed a fully automatic workflow for whole breast irradiation using tangent hybrid IMRT. The auto planning technique kept all the desirable features of manual planning including tangent beam setup, field‐in‐field techniques with the majority of dose delivery by open beams, skin flash, ability to use mixed energies, beam weight optimization and heart and lung blocks. In hybrid IMRT planning, prescribing to max point dose in the 3D prescription allowed the maximum percentage of dose to be delivered by open beams while improving robustness and preventing IMRT segments from being placed at the skin surface. Replacing the manually created segments with inversely planned IMRT, although increased plan MU slightly, allowed auto plans to significantly improve the homogeneity of dose in the breast and reduce dose to lungs and heart. However, the use of IMRT as part of the auto planning process may increase the costs of treatment (slightly increasing treatment time) and must be weighed against improvements in treatment planning quality and efficiency. Whether or not insurance companies consider this hybrid plan as an IMRT plan or 3D plan is beyond the scope of this study.

In breast IMRT planning, the choice of the maximum number of segments is crucial as it affects the plan quality, delivery efficiency, and plan robustness to motion. Purdie et al. chose the maximum number of segments between 6 and 12 based on the target volume.^4^ We arbitrarily chose 20 as the maximum number of segments for free breathing patients, and 10 for breath hold patients. To study the impact of segment number, case #1 was planned with 5, 10, 20, and 50 maximum number of segments respectively. Table 6 compares the influence of the maximum number of segments to the plan quality, plan MUs, and beam delivery time. For as few as five segments, the auto plan showed better plan quality compared with the manual plan. Increasing the number of segments does not reduce lung or heart dose because beam geometry was the determining factor for lung and heart sparing. For segment number <20, increasing the maximum segment number reduced hot spot without increasing plan MU. However, although plan MU is almost independent of the segment number, more segments increased delivery time. For breath hold patients, shorter delivery time is desirable. Therefore, a maximum of 20 segments were used for free breathing patients to maximize the dose homogeneity, and a maximum of 10 segments were used for breath hold patients to improve efficiency with a slight compromise in dose homogeneity.

**Table 6:** The influence of the maximum number of segments to plan quality, MU and delivery efficiency of automatic breast intensity modulated radiation therapy (IMRT) planning.

	PTVeval_Breast V95/%	PTVeval_Breast V105/%	Ipsi Lung V20/%	Heart Dmean/Gy	Total MU	Delivery time/s
Clinical plan (3 segments)	93.5	17.7	14.7	1.0	312	43
Auto plan (5 segments)	93.1	7.7	12.2	0.8	320	41
Auto plan (10 segments)	93.3	4.7	12.1	0.8	332	57
Auto plan (20 segments)	93.2	2.3	12.1	0.8	328	82
Auto plan (50 segments)	93.2	3.1	12.1	0.8	339	112

**Figure 5:**
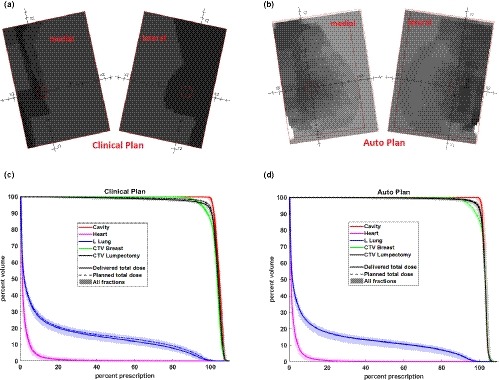
Comparison of beam fluence pattern (top figures) and delivered dose volume histogram (DVH) (bottom figures) with the measured patient motion for case #1 between the clinical (left figures) and auto (right figures) plans. In the DVH figures, the dashed lines represent the planned DVH, the shaded regions represent delivered DVHs with the measured patient motion for each treatment fraction, and the solid lines represent the total delivered DVH of all fractions.

Another common question for breast IMRT is whether the use of intensity modulated beams will compromise plan robustness and increase sensitivity to the patient motion. To explore this question, for an example patient, we simulated the delivered dose in the presence of motion for the auto plan (with maximal 20 segments) and the clinical plan. Case #1 was treated with active breath hold using ABC (active breathing coordinator, Elekta, Stockholm, Sweden) and monitored with the AlignRT system (VisionRT Ltd., London, UK). The delta of the real‐time patient surface compared with the reference skin surface extracted from the CT images was recorded during the treatment. We extracted the delta at the time of the delivery for each beam in every fraction and calculated the delivered dose for each fraction. Throughout the treatment course, the delta in vertical, longitudinal, lateral, roll, yaw and pitch ranged from 0.1–5.9 mm, −1.6–3.4 mm, −2.4–2.8 mm, −1–1.4°, −1.6–1.3° and −1.0–2.6° respectively. Figure 5 compares the simulated delivered dose of each fraction, the total delivered dose of all fractions and the planned dose for auto and clinical plans. DVHs to the lumpectomy cavity, CTV_breast, CTV_Lumpectomy, ipsilateral lung, and heart were compared. Both clinical and auto plans were robust to the patient motion. The auto plan showed slightly better agreement between planned and delivered total dose to CTV_Lumpectomy and ipsilateral lung. A possible reason may be that auto plan created fluence pattern smoother than the clinical plan, as shown in Fig. 5.

Auto‐contouring targets and normal structures is a challenging task in radiation therapy. For a fully automatic treatment planning workflow, the accuracy of auto contours is crucial as it affects beam setup, plan optimization, and plan evaluation. In this study, we compared auto contours with clinically approved manual contours for all patients and reported mean dice coefficients of 0.84, 0.98, and 0.94 for CTV_Breast, lungs, and heart, respectively. This result was comparable with previous publications: Eldesoky et al.^5^ reported mean dice coefficients of 0.86, 0.97 and 0.92 and Velker et al.^6^ reported mean dice coefficients of 0.88, 0.97 and 0.90 for breast, lungs, and heart, respectively.

To further evaluate the impact of the auto‐contouring errors on automatic breast treatment planning, for an example patient (case #1), Table 7 compares the plan parameters, dose distribution, and plan quality between the auto plan using auto contours and the auto plan using clinical contours. For this particular patient, the Dice coefficients between auto contours and clinical contours were 0.80, 0.98, and 0.92 for CTV_breast, lungs, and heart, respectively. Although target and normal structure volumes varied, the plan parameters (beam gantry and collimator angles, jaw sizes, and plan MU) were similar between the two plans. The 90% and 50% isodose volumes of the two plans overlapped by 94% and 95% respectively. DVH metrics to targets and normal structures (evaluated using clinical contours) were comparable between the two plans. The auto planning technique introduced in this study is robust to the errors of auto‐contouring. Two main reasons may explain the insensitivity of the auto plan to contouring errors: (a) the auto‐contoured normal structures closely matched the clinically approved manual contours (>0.9 dice coefficients); (b) we applied restrictions in beam geometry optimization: beam gantry and collimator angles cannot deviate from initial angles by more than 10° and beams were not allowed to cross midline.

**Table 7:** Comparison of the auto plan using auto contours with the auto plan using clinical contours.

	Auto plan w auto contours		Auto plan w clinical contours
Energy	10X		10X
Gantry/°	313		311
Collimator/°	10		8
X1 (posterior jaw)/cm	4.5		4.5
Y1 (inferior jaw)/cm	9.7		9.5
Y2 (superior jaw)/cm	11.6		11
Plan MU	326		332
Dice coefficient of 90% IDL	0.94		
Dice coefficient of 50% IDL	0.95		
Plan quality	DVH to auto contours	DVH to clinical contours	DVH to clinical Contours
PTVeval_Breast V95/%	85.8	92.7	93.3
PTVeval_Lumpectomy V95/%	97.1	97.4	96.4
PTVeval_Breast V105/%	6.1	5.6	4.7
Lt Lung V20/%	12.3	12.2	12.1
Heart Dmean/Gy	0.7	0.8	0.8

It is worth noting that although the plan parameters and dose distribution were robust to contour variations, DVH metrics were sensitive to how the targets and organs at risk were contoured. As shown in Table 7, PTVeval_Breast V95 from the auto plan was lower when evaluated with the auto contour than with the clinical contour. As shown in Fig. 2, the auto‐segmented CTV_Breast and PTVeval_Breast was larger than the manual contours. The heart mean dose was slightly lower when evaluated with the auto contour than with the clinical contour, again due to smaller auto‐contoured heart than the clinical contour. Therefore, it is still recommended to review and edit the auto contours for plan evaluation.

## 5 CONCLUSIONS

An automated treatment planning technique was developed for whole breast irradiation using hybrid IMRT. Compared with manual planning, auto planning improved planning efficiency and plan quality. A future study will focus on the assessment of the robustness of auto plans with more patient data.

## CONFLICT OF INTEREST

Dr. Chirag Shah received a research grant from VisionRT, Inc. outside of this study. Dr. Ping Xia received a grant from Philips Healthcare outside of this study. Dr. Chirag Shah also receives consultation fees from Impedimed and Varian Medical Systems.
